# A novel model for malaria prediction based on ensemble algorithms

**DOI:** 10.1371/journal.pone.0226910

**Published:** 2019-12-26

**Authors:** Mengyang Wang, Hui Wang, Jiao Wang, Hongwei Liu, Rui Lu, Tongqing Duan, Xiaowen Gong, Siyuan Feng, Yuanyuan Liu, Zhuang Cui, Changping Li, Jun Ma

**Affiliations:** Department of Health Statistics, College of Public Health, Tianjin Medical University, Heping District, Tianjin, P.R. China; Politechnika Krakowska im Tadeusza Kosciuszki, POLAND

## Abstract

**Background and objective:**

Most previous studies adopted single traditional time series models to predict incidences of malaria. A single model cannot effectively capture all the properties of the data structure. However, a stacking architecture can solve this problem by combining distinct algorithms and models. This study compares the performance of traditional time series models and deep learning algorithms in malaria case prediction and explores the application value of stacking methods in the field of infectious disease prediction.

**Methods:**

The ARIMA, STL+ARIMA, BP-ANN and LSTM network models were separately applied in simulations using malaria data and meteorological data in Yunnan Province from 2011 to 2017. We compared the predictive performance of each model through evaluation measures: RMSE, MASE, MAD. In addition, gradient-boosting regression trees (GBRTs) were used to combine the above four models. We also determined whether stacking structure improved the model prediction performance.

**Results:**

The root mean square errors (RMSEs) of the four sub-models were 13.176, 14.543, 9.571 and 7.208; the mean absolute scaled errors (MASEs) were 0.469, 0.472, 0.296 and 0.266 and the mean absolute deviation (MAD) were 6.403, 7.658, 5.871 and 5.691. After using the stacking architecture combined with the above four models, the RMSE, MASE and MAD values of the ensemble model decreased to 6.810, 0.224 and 4.625, respectively.

**Conclusions:**

A novel ensemble model based on the robustness of structured prediction and model combination through stacking was developed. The findings suggest that the predictive performance of the final model is superior to that of the other four sub-models, indicating that stacking architecture may have significant implications in infectious disease prediction.

## Introduction

Malaria is an acute parasitic infection caused by plasmodium, which is mainly transmitted through mosquitoes. Although many countries have made remarkable progress towards eliminating malaria, the illness remains a serious public health issue, with an estimated 219 million cases globally in 2017[1]. Therefore, the World Health Organization (WHO) launched the WHO global technical strategy for malaria from 2016–2030 to accelerate progress towards eliminating malaria [2]. In the early 1970s, malaria was still one of the most common infectious diseases, with 2.4 million cases in mainland China. After decades of constant efforts at the national level, the epidemic of malaria has been effectively controlled in most parts of China. Moreover, the number of cases of malaria decreased from 62124 cases in 1994 to 25064 cases in 2015. To achieve the goal of eliminating malaria, overcoming the technical difficulties of malaria prediction models and surveillance systems is of utmost importance to researchers and requires considerable attention.

There is growing interest in using malaria prediction models to help clinical and public health services strategically implement prevention and control measures. Previously, studies that predicted malaria incidence mainly adopted linear regression [3], Poisson regression [4], Spearman’s correlation [5], non-linear methods [6], and autoregressive integrated moving average models (ARIMAs) [7]. Due to the transmissibility and seasonality of malaria, models with an ARIMA structure have more predictive power compared to other methods [8]; such models have become conventional models and used to applied to predict malaria and other numerous infectious diseases with similar periodic patterns over the past decades [9–10]. However, the main limitation of ARIMA models is that linear correlation structures are assumed for the time series data, and actual time series data do not always satisfy the assumption [11–13]. Shi et al. noted that deep learning yields better prediction performance than ARIMA models [14]. A deep learning algorithm can greatly improve the prediction performance when manipulating irregular and non-stationary time series data because it can discover and characterize the complex structural features of a data set [15]. A back-propagation (BP) neural network is one of the most widely used traditional methods in deep learning. Lee et al. showed that BP neural networks can be used to model meteorological factors and malaria data because of their distinct advantages, such as timely learning, strong comprehensive ability, and low data requirements [16]. In addition, among all the emerging deep learning architectures, the long short-term memory network (LSTM) has been the most successful in processing and predicting events with relatively long intervals and lags in time series [17]. Sangwon et al. found that prediction performance could be improved using LSTM networks [18].

As discussed above, deep learning methods are useful for establishing effective models in the field of predicting infectious diseases. However, with only a single prediction model, the prediction accuracy may be limited, even with optimum parameters. Hence, a novel approach called stacked generalization has attracted considerable attention. Stacked generalization using stacking frameworks with various machine learning methods can be applied in meta-models to maximize the prediction performance. Bhatt et al. proposed a robust model that exhibited better prediction performance than sub-models in the field of predicting the malaria prevalence [19]. However, no studies have applied stacking architectures or LSTM algorithms for malaria prediction.

In this study, based on the results of a spatiotemporal analysis of malaria cases in mainland China from 2007 to 2017, prediction models of malaria are specified for provinces with malaria epidemics. The purpose of this study is to compare the performance of traditional time series models and deep learning algorithms in malaria case prediction and to explore the application value of stacking algorithms in malaria case prediction.

The rest of this paper is organized as follows. Section 2 describes the data sources used in this study and introduces the analysis methodology used to design the prediction model. Results from real data sets and their implications are reported in Section 3. Section 4 discusses the limitations. Section 5 contains the concluding remarks.

## Materials and methods

### Data sources

Monthly reports of malaria cases in mainland China from 2007 to 2017 were obtained from the National Scientific Data Sharing Platform for Population and Health. Visual analysis was conducted using data from 2007 to 2016. In 2010, 13 ministries and commissions of the Chinese government jointly issued the “China Malaria Elimination Action Plan (2010–2020)”, which proposed to eliminate malaria in most parts of China by 2015 and achieve the goal of eliminating malaria by 2020 [20]. Then, the malaria cases in Yunnan Province from 2011 to 2017 were used for modelling. In addition, because malaria is a climate-sensitive infectious disease, a series of studies predicted the incidence of malaria based on meteorological factors and showed that a warming climate may potentially increase the risk of malaria transmission [21]. Moreover, previous studies also indicated that malaria incidence prediction could be more effective if climate data are used [22]. Hence, in this study, meteorological data were incorporated into the prediction models. The meteorological data used in this study were obtained from the National Meteorological Information Center (http://data.cma.cn/). The hourly meteorological data were collected at national surface stations throughout China and include data on the temperature, relative humidity, air pressure, vapor pressure, moisture level, wind velocity, precipitation, sunshine duration, and days with daily precipitation > = 0.1 mm.

**Fig 1:**
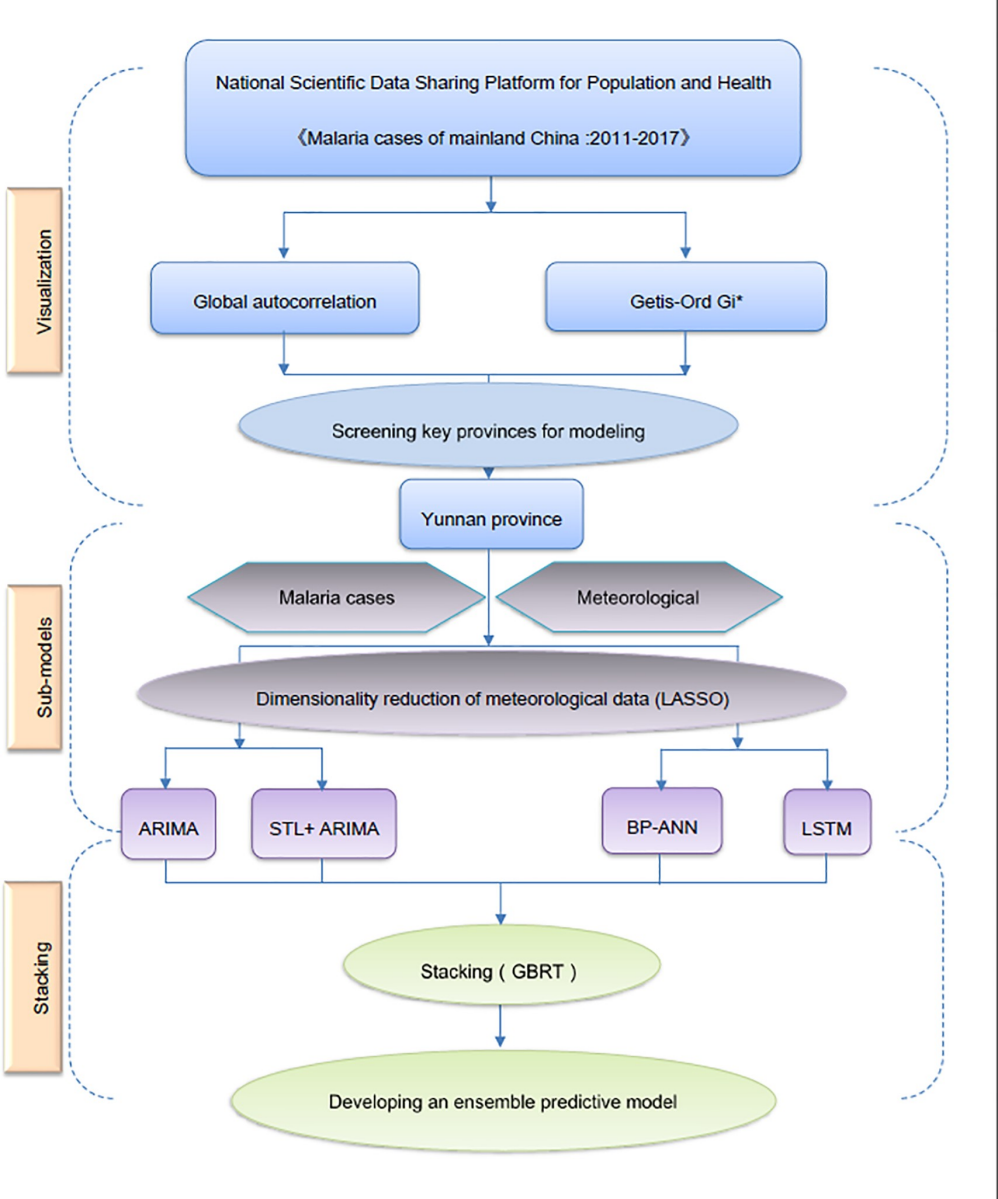
Flow chart of this study.

Fig 1 shows the overall framework of this study, including a visual analysis of the data, the sub-models (the ARIMA model, the STL+ARIMA model, the BP neural network (BP-ANN), and the LSTM network) and the process of stacked generalization.

## Methods

In the modelling process, the data from 2011 to 2015 were used as the training set (n = 60), the data from 2016 were used as the verification set (n = 12), and the number of malaria cases in 2017 was used as the test set (n = 12). In the process of stacking, the data from 2011 to 2016 were cross-validated to predict the number of malaria cases in 2017. The ranges considered for each parameter are listed in S1 File.

### Traditional time series model: ARIMA

The ARIMA model was obtained after introducing an integrated difference concept into the ARMA model [23]. The basic concept of the ARIMA model is to treat the data sequence for a given variable over time as a random sequence and use a mathematical model to fit the time series data [22]. Once this model is identified, it can predict future values from past and present values of the time series. First, the ARIMA model assesses a stationary sequence through the results of a unit root test. For stationary time series data, the ARIMA model can be an effective analysis method. In addition, the ARIMA model can also be applied to manipulate non-stationary data after differentiating the non-stationary time series to achieve a stationary state [18]. Because the malaria case data are seasonal, this study adopts the seasonal ARIMA model, which can be expressed as ARIMA(p, d, q)(P, D, Q)s, where p is the order of autoregression, d is the order of the differentiation, and q is the order of the MA. (P, D, Q) is the order of the seasonal portion of the function, and s is the seasonal period.

The formula for the seasonal ARIMA model is written as follows:

$$\nabla^{d}\nabla_{s}^{D}x_{t}=\frac{\Theta (B)\Theta_{s}(B)}{\Phi (B)\Phi_{s}(B)}\varepsilon_{t}$$

$$\Theta (B)=1-\theta_{1}B-\cdots -\theta_{q}B^{q}$$

$$\Phi (B)=1-\phi_{1}B-\cdots -\phi_{q}B^{q}$$

$$\Theta_{s}(B)=1-\theta_{1}B^{S}-\cdots -\theta_{Q}B^{QS}$$

$$\Phi_{s}(B)=1-\phi_{1}B^{S}-\cdots -\phi_{Q}B^{PS}$$

where *x*_*t*_ is the actual value at time t, *ε*_*t*_ is an independent disturbance, B is the backshift operator, Θ(*B*) is the autoregressive operator, Φ(*B*) is the MA operator, and Θ_*s*_(*B*) and Φ_*s*_(*B*) are the seasonal operators of the model.

### Traditional time series model: Seasonal and trend decomposition using loess (STL+ARIMA)

The STL+ARIMA model decomposes time series data with seasonal factors into trend factors, seasonal factors and random factors. Notably, trend factors can capture long-term changes, seasonal factors can capture cyclical changes within a year, and random factors can capture changes that cannot be explained by trends or seasonal effects [24]. Thus, the STL method can be applied to can analyse any type of seasonal trend, and the seasonal trend can change over time. In the model establishment process, the seasonality is predicted using the seasonal naïve method. For seasonal adjustment, the non-seasonal ARIMA model is used in this study [25]. This model can be expressed as follows:

$$x_{t}=T_{t}+S_{t}+I_{t}$$

where T_*t*_ represents the trend factor, S_*t*_ represents the seasonal factor, and I_*t*_ represents the random factor.

### Deep learning algorithm: Back-propagation neural network (BP-NN)

An artificial neural network (ANN) is a mathematical model that simulates the human brain to solve problems, and a BP neural network is one of the most widely used neural network models. BP-NNs are multilayer feedforward NNs trained based on the error back-propagation algorithm. The learning rule of a BP-NN is to adopt the steepest descent method to continuously adjust the weights by calculating the gradient of the loss function [26]. The final optimal model is obtained when the sum of the squared error of the network reaches a minimum. In this study, a BP-NN with a single hidden layer is used to predict the number of malaria cases, and the nnetar {forecast} function in R 3.5.1 software is used to establish the network.

A single hidden layer feedforward network is the most widely used network form for time series modelling and forecasting [27]. The model is characterized by a network of three layers of simple processing units connected by acyclic links. The relationship between the output (*y*_*t*_) and the inputs (*y*_*t*−1_; *y*_*t*−2_; …; *y*_*t*−*p*_) is based on the following mathematical representation:

$$y_{t}=\alpha_{0}+\sum_{j=1}^{q}\alpha_{j}g\left(\beta_{0j}+\sum_{i=1}^{p}\beta_{ij}y_{t-i}\right)+\varepsilon_{t}$$

where *α*_*j*_(j = 0, 1, 1, …, q) and *β*_*ij*_ are the model parameters, often called the connection weights; p is the number of input nodes; and q is the number of hidden nodes.

### Deep learning algorithm: LSTM network

**Fig 2:**
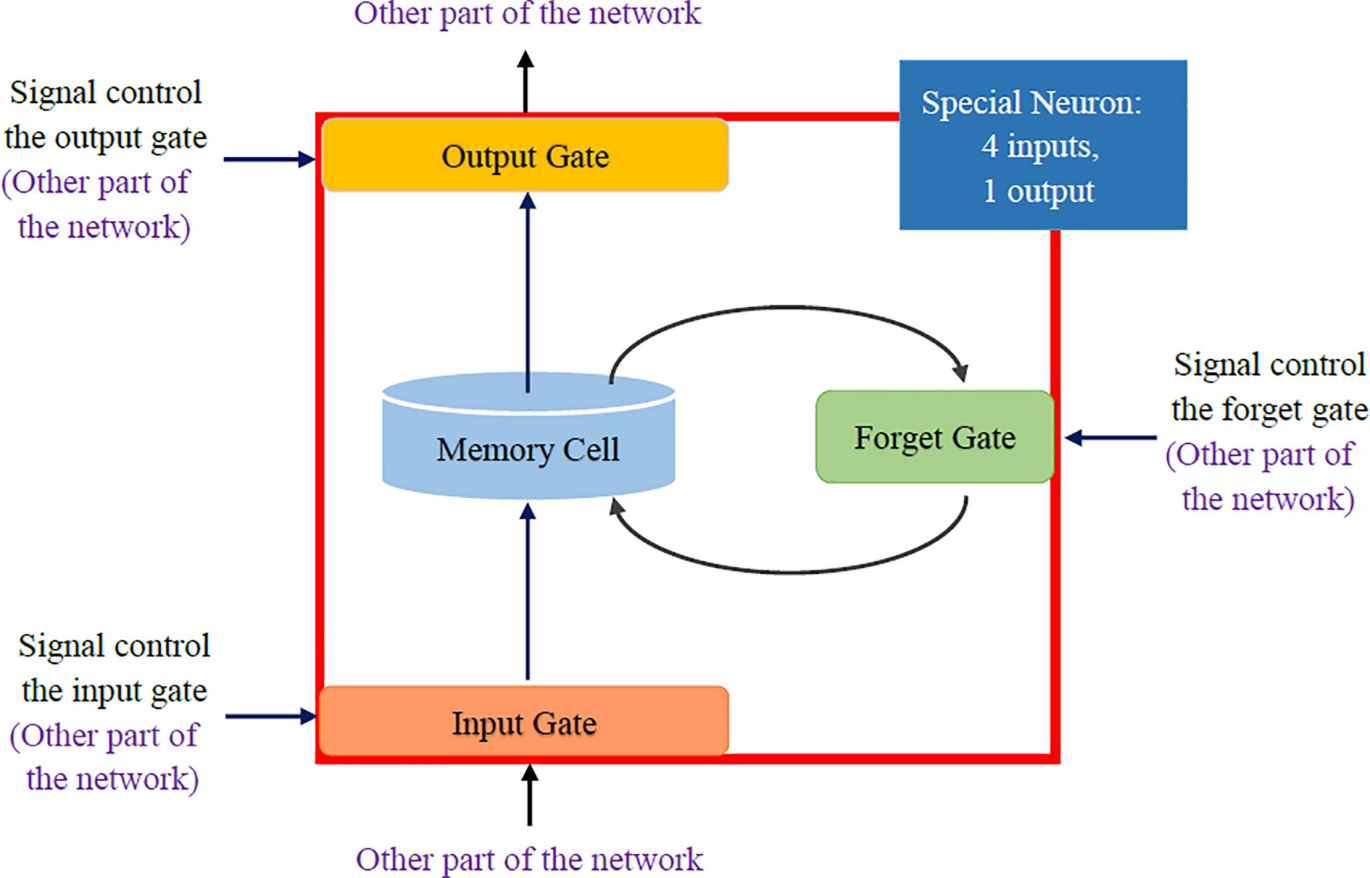
The framework of LSTM.

An LSTM network is a temporally recurrent neural network (RNN) that uses a BP algorithm for network training and is suitable for processing and predicting events with relatively long intervals and delays in time series [27]. The most common LSTM architecture includes a memory storage unit and three gates, including a forget gate, an input gate and an output gate. The hidden layer in the traditional RNN model is replaced with a memory storage unit to process vanishing and exploding gradient problems [28]. As shown in Fig 2, the LSTM gates are activated through a sigmoid function. Intuitively, the input gate determines the degree to which a new value flows into the memory storage unit. Additionally, the forget gate determines the degree to which a value remains in the unit, and the output gate determines the degree to which the value in the unit is used in the activation of the LSTM network [22]. The equations for forgetting, storing, renewing, and outputting information in the cell are shown below:

$$f_{t}=\sigma (\omega_{f}.[h_{t-1},x_{t}]+b_{f})$$

$$i_{t}=\sigma (W_{i}.[h_{t-1},x_{t}]+b_{i})$$

$${\tilde{C}}_{t}=tanh(W_{C}.[h_{t-1},x_{t}]+b_{C})$$

$$C_{t}=f_{t}\times C_{t-1}+i_{t}\times {\tilde{C}}_{t}$$

$$o_{t}=\sigma (W_{o}.[h_{t-1},x_{t}]+b_{o})$$

$$h_{t}=o_{t}\times tanh(C_{t})$$

where *x*_*t*_ is the input vector for the LSTM unit, *f*_*t*_ is the activation vector of the forget gate, i_t_ is the activation vector of the input gate, *o*_*t*_ is the activation vector of the output gate, *h*_*t*_ is the output vector of the LSTM unit, C_t_ is the cell state vector, σ is the sigmoid function, W is the weight matrix, b represents the bias vector parameters, and *h* is the number of hidden units.

In this study, an LSTM architecture with two hidden layers is used to predict the cases of malaria, and the “keras” package in R 3.5.1 software is used to establish the NN.

### Stacking

**Fig 3:**
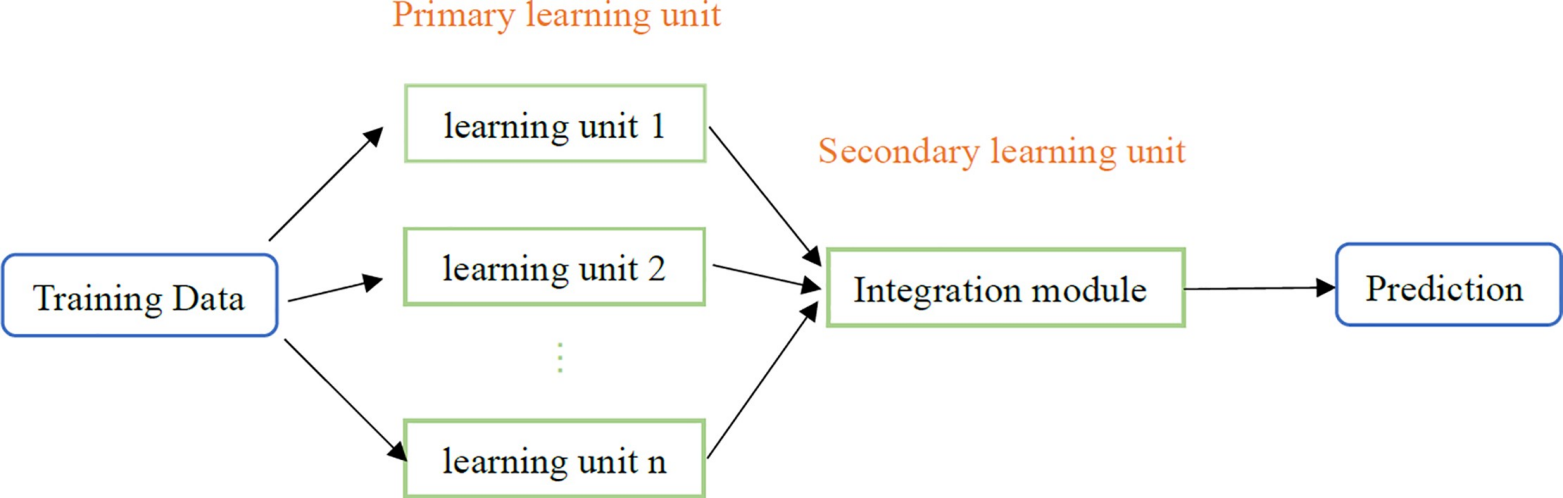
The framework of stacking.

Ensemble learning is a technical framework that uses multiple learning algorithms to complete learning tasks and obtain better predictive performance than that obtained from any individual learning algorithm [29–31]. According to the variety of ensemble methods, current ensemble learning can be roughly divided into three categories: bagging, boosting and stacking [32]. Unlike stacking, bagging and boosting can only combine the same type of machine learning algorithm [33]. A stacking approach can reduce the generalization error by training a meta-learning algorithm to combine the predictions of several distinct primary learning algorithms [32]. First, the primary learning algorithms are trained with the original training set, and a secondary model is then trained based on the prediction results of the primary learning algorithms. For example, the simplest secondary model is to simply vote on the results of multiple primary learning machines. Stacking depends on the differences among the learning algorithms to obtain diverse learning, and secondary models can be assessed to best integrate different learning methods and obtain optimal prediction results. Compared with bagging and boosting, stacking tends to have higher prediction precision and lower risk of overfitting [34]. In this study, a complex architecture that combines both a traditional time series model and deep learning is proposed to take advantage of the unique strengths of these methods in linear and nonlinear modelling. The prediction performance of the stacking ensemble method is better than that of baseline models with a combined learning approach (voting method or averaging method) [35]. Fig 3 is the Stacking framework. In this study, gradient-boosted regression trees are used as the ensemble predictors to combine the other four sub-models, and the “rpart” package in R 3.5.1 software is implemented to establish the model.

### Forecasting accuracy measures

The root mean square error (RMSE), mean absolute scaled error (MASE) and mean absolute deviation (MAD) were selected as the evaluation indicators to measure the precision and performance of the prediction models. RMSE is a measure of the deviation between values predicted by a model and the actual values [36]. Generally, a high RMSE reflects poor performance, and vice versa. However, comparisons between values at different scales are invalid because this measure is directly dependent on the corresponding error [30]. Thus, the RMSE can be expressed as follows:

$$RMSE=\sqrt{\frac{\sum ({\hat{y_{t}}-y_{t})}^{2}}{n}}$$

where $\hat{y_{t}}$ is the predicted value, *y*_*t*_ is the actual observed value, and n is the quantity of observations.

Based on the benchmark prediction method, MASE scales the absolute mean error in a sample, which is clearly independent of the scale of the data [36]. If a proportional error is derived from a prediction that is better than that for the average one-step baseline prediction, then the proportional error is less than 1; otherwise, the value is greater than 1. The MASE can be simply formulated as follows:

$$MASE=mean(|q_{j}|)$$

$$q_{j}=\frac{e_{j}}{\frac{1}{T-m}\sum_{t=m+1}^{T}\left|y_{t}-y_{t-m}\right|}$$

where *e*_*j*_ is the difference between the predicted value and the actual value at time j, *y*_*t*_ is the actual value at time t, m is the seasonal period and *y*_*t*−*m*_ is the actual value at time t-m.

Mean Absolute Deviation (MAD) is the average of the absolute values of the deviations of all individual observations from the arithmetic mean. MAD can avoid the problem that the errors cancel each other out, so it can better provide measures of expected deviation of a random variable from its mean value.

$$MAD=\frac{1}{n}\sum_{1}^{n}\left|(\hat{y_{t}}-y_{t})-m(\hat{y_{t}}-y_{t})\right|$$

where $\hat{y_{t}}$ is the predicted value, *y*_*t*_ is the actual observed value, and n is the quantity of observations, $m(\hat{y_{t}}-y_{t})$ is the mean of the difference.

## Results and discussion

According to the results of spatiotemporal analysis and a Getis-Ord Gi* hotspot analysis of malaria cases in mainland China from 2007 to 2016 (see S1 and S2 Figs), Yunnan Province was selected as a key province for subsequent modelling. In addition, the malaria cases in Yunnan Province were modelled considering meteorological data.

Least absolute shrinkage and selection operator (LASSO) regression was adopted for the dimension reduction of the meteorological variables due to the correlations among meteorological factors, which can affect the predictive performance of traditional times series models (see S3 Fig). Although deep learning algorithms have no requirement regarding the restriction of the correlations among variables, the sub-models should adopt consistent meteorological variables for comparability. Four meteorological variables were selected, including the sunshine duration, average temperature, average wind speed and precipitation, according to the results of dimension reduction based on the model of Yunnan Province.

The time series plot (see S4 Fig) suggests that malaria cases decreased rapidly from 2011 to 2012, followed by a relatively stable period from 2013 to 2017. According to the results of the unit root test, the sequences were stationary (Dickey-Fuller = -5.4227, *P* = 0.01). S5 Fig shows the seasonal trend decomposition plot of the malaria cases in Yunnan Province from 2011 to 2017. The cases of malaria exhibited a significant seasonal trend in Yunnan Province. In addition, a few strong peaks can be identified in the summer period (June-August).

In this study, all modelling was implemented via R 3.5.1, as noted earlier, and each model was built using distinct statements. We attempted to genuinely evaluate the prediction performance of the proposed model and all sub-models. The RMSE, MASE and MAD were selected as the measures of forecasting performance. Table 1 gives the prediction results for the malaria cases.

**Table 1:** Results of model comparison.

Index	Method				
	ARIMA	STL+ARIMA	BP-ANN	LSTM	Stacking
**RMSE**	13.176	14.543	9.571	7.208	6.810
**MASE**	0.469	0.472	0.297	0.266	0.224
**MAD**	6.403	7.658	5.871	5.691	4.625

This study used the auto.arima statement to build the SARIMA model, in which lag = 12. The optimal ARIMA model for Yunnan Province is (0,1,2) × (0,1,1) [12]. Moreover, the seasonal decomposition model was implemented using the stlf statement, and the optimal model is ARIMA (0,1,2). In addition, by using the nnetar statement, the BP-ANN model was established, and the optimal parameter set is "NNAR (12,1,3) [12] ". In the LSTM used in this study, the optimizer is RMSprop, the learning rate is 0.001, the loss function is MSE, and the number of hidden layers is 2. Table 1 shows that in the four basic models, the prediction performance of the LSTM network is better than that of the other three models. Among these sub-models, the RMSE of the LSTM algorithm decreased by 45.3%, 50.4% and 24.7% compared with those of ARIMA, STL+ARIMA and BP-ANN methods, respectively. Additionally, the MASE of the LSTM model was 0.266, which was lower than the MASE values of the other sub-models. In terms of MAD, the percentage improvements of the LSTM over the ARIMA, STL+ARIMA and BP-ANN were 11.12%, 25.69% and 3.07%, respectively.

In this study, GBRT was used as an integrated model to stack the four sub-models. As shown in Table 1, the prediction performance of the model improved after stacking. The RMSE of the model is 6.810, a decrease of 48.3%, 53.2%, 28.9% and 5.5% compared to the RMSEs of the other four sub-models. The MASE of the integrated model decreased by 52.2%, 52.5%, 24.58% and 15.8% compared to the values for the other four sub-models. Additionally, the ensemble model has a MAD value of 4.625, which was much lower than above sub-models. The empirical results clearly suggest that the SABL-Stacking model outperforms each sub-model separately.

**Fig 4:**
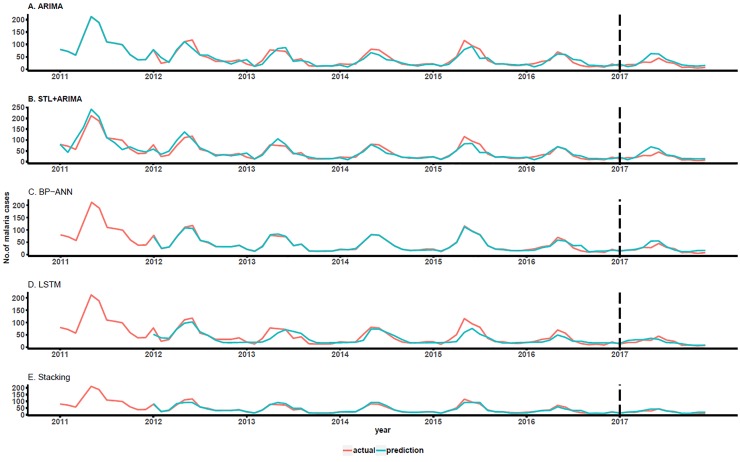
The prediction graph of each model.

To make the prediction results more intuitive, a prediction graph of the number of malaria cases for each model from 2011–2017 was drawn. A point-to-point comparison of the predicted and actual values can be seen in Fig 4. As shown, the predicted values of the model generally agree with the actual values. Although for some data points, the LSTM sub-model yields worse predictions than the ARIMA, STL+ARIMA or BP-ANN model, the overall forecasting capability significantly improved. The prediction effect of the stacking model is significantly higher than those of individual models, and the gap between the predicted and actual values decreased.

## Discussion

The ARIMA model, seasonal decomposition model, and BP-NN have been widely used in the field of infectious disease prediction in previous studies. Ebhuoma et al. used the SARIMA model to predict the incidence of malaria in KwaZulu-Natal, South Africa, and the results provided useful theoretical support for malaria control and elimination in this region [13]. Chena et al. used ANNs combined with meteorological data to model and predict the oyster norovirus outbreak in the Gulf of Mexico in 1996–2010, and the performance of the proposed prediction model was better than that of traditional models [37]. In addition, as a developmental form of RNNs in the field of deep learning, LSTM networks have successfully applied for handwriting recognition, speech recognition and a wide range of NLP tasks, such as machine translation, constituency parsing, language modelling and natural language inference, including for rule-based systems [38–48]. But the previous studies mentioned above, deep learning algorithms were not used or the LSTM was not considered. In this study, ARIMA, seasonal decomposition, BP-ANN and LSTM models were used to predict the number of malaria cases. Consistent with the results of Chena et al., the performance of BP-ANN is superior to traditional models including ARIMA and seasonal decomposition [37]. The prediction performance of the LSTM sub-model was significantly better than that of the other three models, indicating that the LSTM method is highly applicable in cases of small sample analysis and infectious disease prediction. It is clear that a positive evolution from traditional models to deep learning has occurred, leading to gradual and sustained progress in the field.

Therefore, we assume that an increasing number of methods will integrate results from multiple predictors with the aim of improving the robustness and accuracy of predictions. Murphree et al. applied a stacking mechanism to predict the likelihood of adverse reactions induced by blood transfusions [49]. Pernia-Espinoza et al. applied stacking to predict the three key points of a comprehensive force-displacement curve for bolted joints in steel structures [50]. Stacking provides a natural and effective method of combining various (often conflicting) findings from independent research activities. Any advancements from an emerging algorithm can be easily and timely reflected in a stacking framework. Therefore, in this case, it is appropriate to use a stacking framework, such as a novel deep learning algorithm, to predict the number of malaria cases. In addition, SABL-Stacking exhibited better prediction performance than other sub-models; consequently, stacking is feasible in the field of malaria case prediction and can provide new methodological concepts for predicting other infectious diseases.

### Limitations

Because the number of malaria cases from 2007 to 2017 displayed multistage fluctuations, the practical significance of the prediction model may be weak if the entire time series is used to establish models. Therefore, this study only selected time series data from 2011 to 2017 for subsequent analysis, which may lead to deviations in the accuracy of predictions. Although the ensemble algorithm in this study improved the prediction accuracy, the predictive performance may be further optimized by choosing other feasible prediction models. Hence, it is difficult to state that the most robust stacking framework was established because the algorithms used in this architecture did not cover all prediction models that could be adopted. Moreover, wide ranges of the model parameters in the LSTM algorithm were not considered. If more model parameters are considered, the stacking architecture could potentially improve, thereby increasing the predictive performance. In summary, this study applies the LSTM algorithm and stacking in the field of malaria case prediction, and these models display better prediction performance than do traditional methods. Subsequent studies can attempt to apply this novel analysis method in the prediction of other infectious diseases.

## Conclusions

In conclusion, malaria is a serious public health issue that threatens personal health and can be widely transmitted. In this context, it is very important to predict malaria resurgence and take corresponding measures in mainland China. Therefore, this study was conducted to reduce the public health burden by predicting malaria cases in provinces with malaria epidemics. Moreover, the aim of this study was to establish a site-specific prediction model that is more robust than existing models. The proposed model, denoted as SABL-Stacking, is an ensemble architecture of deep learning algorithms and traditional time series models. Remarkably, the prediction performance observed in this paper indicates that SABL-Stacking's prediction performance is superior to traditional time series models (ARIMA, seasonal decomposition) and better than deep learning algorithms (BP-ANN and LSTM); therefore, SABL-Stacking model can be effectively applied in the malaria forecasting field. The next step in our research plan is to investigate the performance of SABL-Stacking for other big data samples, especially in the field of infectious diseases.

## Supporting information

S1 Fig Spatiotemporal analysis from 2007 to 2010. (PDF)

S2 Fig Spatiotemporal analysis from 2011 to 2016. (PDF)

S3 Fig Variable selection. (PDF)

S4 Fig Time series plot. Monthly number of malaria cases from 2011 to 2017.
(PDF)

S5 Fig Seasonal trend decomposition chart. (PDF)

S1 File Parameter setting. (XLSX)

## Abbreviations

ARIMA: autoregressive integrated moving average
STL+ARIMA: seasonal and trend decomposition using Loess
BP-ANN: back-propagation artificial neural network
LSTM: long short-term memory
GBRT: gradient-boosted regression trees
RMSE: root mean square error
MASE: mean absolute scaled error
MAD: mean absolute deviation
RNN: recurrent neural network
LASSO: least absolute shrinkage and selection operator
