# Supplementary figures and images for: A novel model for malaria prediction based on ensemble algorithms
Source: PLoS One. 2019 Dec 26;14(12):e0226910. doi: 10.1371/journal.pone.0226910 (PMC6932799; doi:10.1371/journal.pone.0226910)

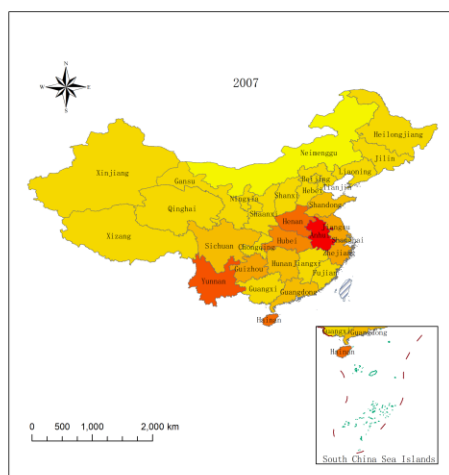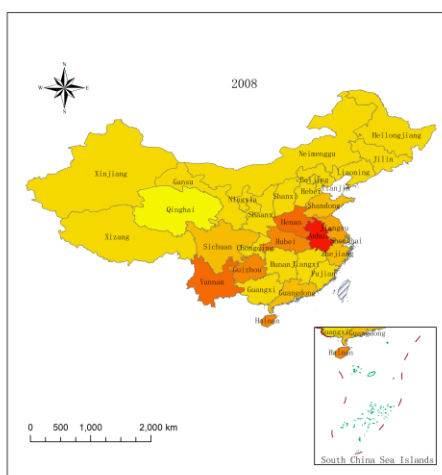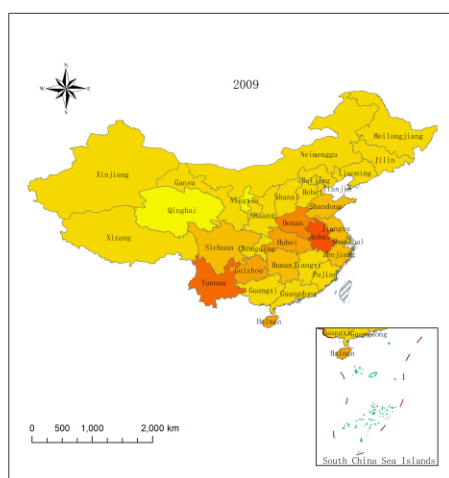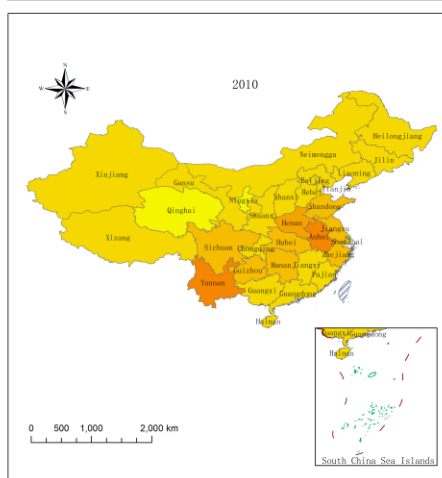

### Graphic Symbol

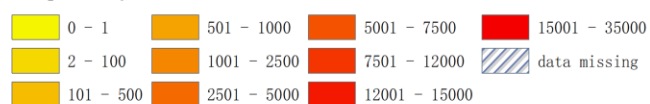

Supplement: S1 Fig — (PDF) [file pone.0226910.s001.pdf]

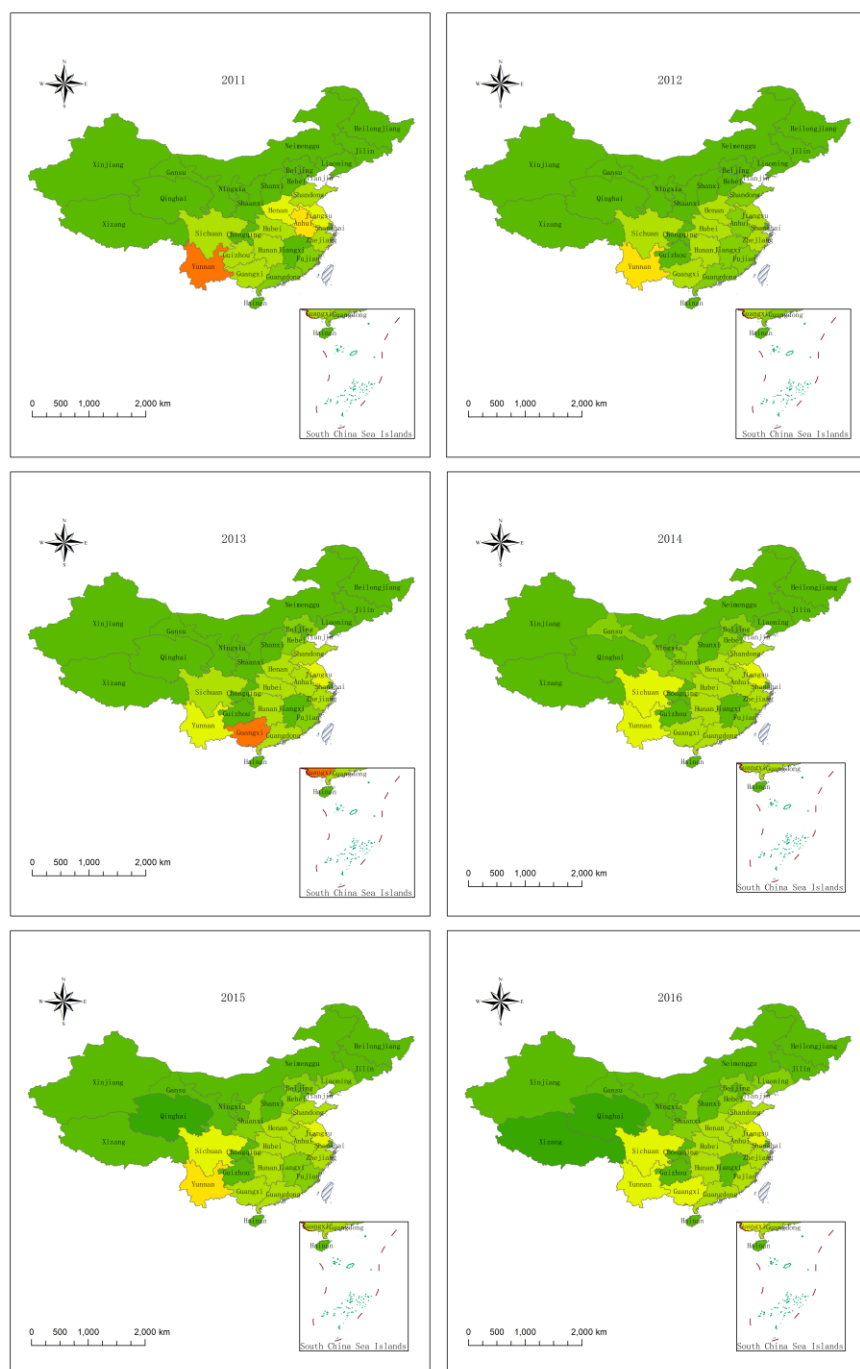

### Graphic Symbols

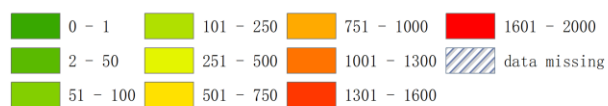

Supplement: S2 Fig — (PDF) [file pone.0226910.s002.pdf]

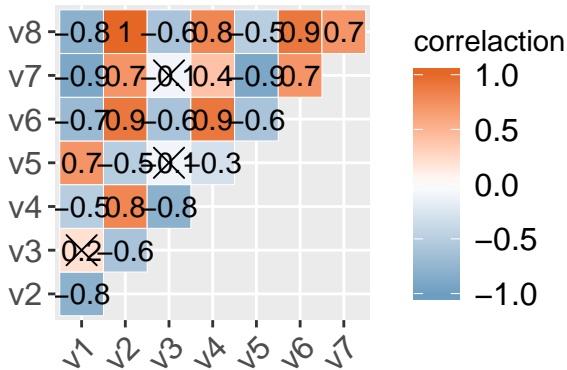

Supplement: S3 Fig — (PDF) [file pone.0226910.s003.pdf]

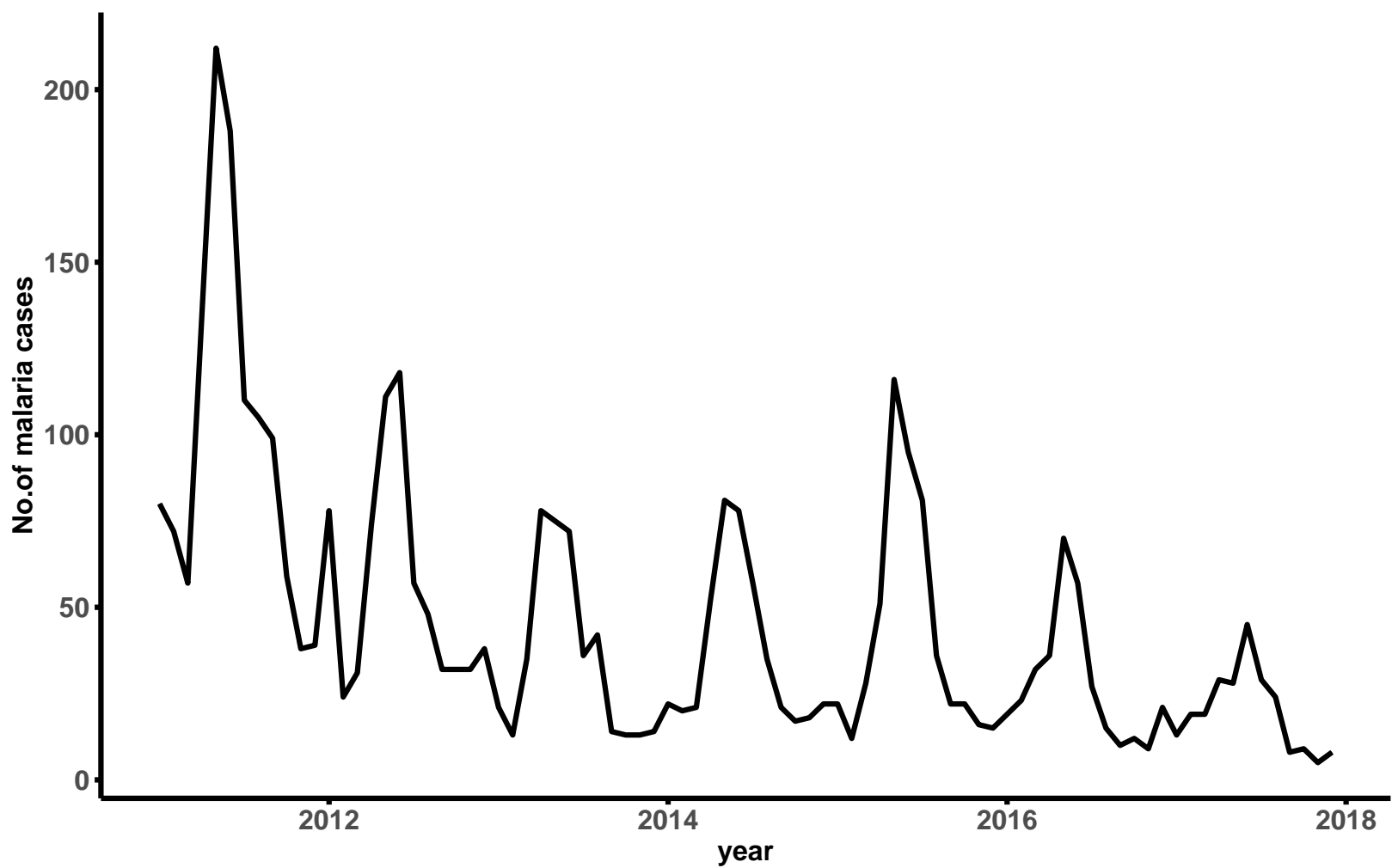

Supplement: S4 Fig — Monthly number of malaria cases from 2011 to 2017. (PDF) [file pone.0226910.s004.pdf]

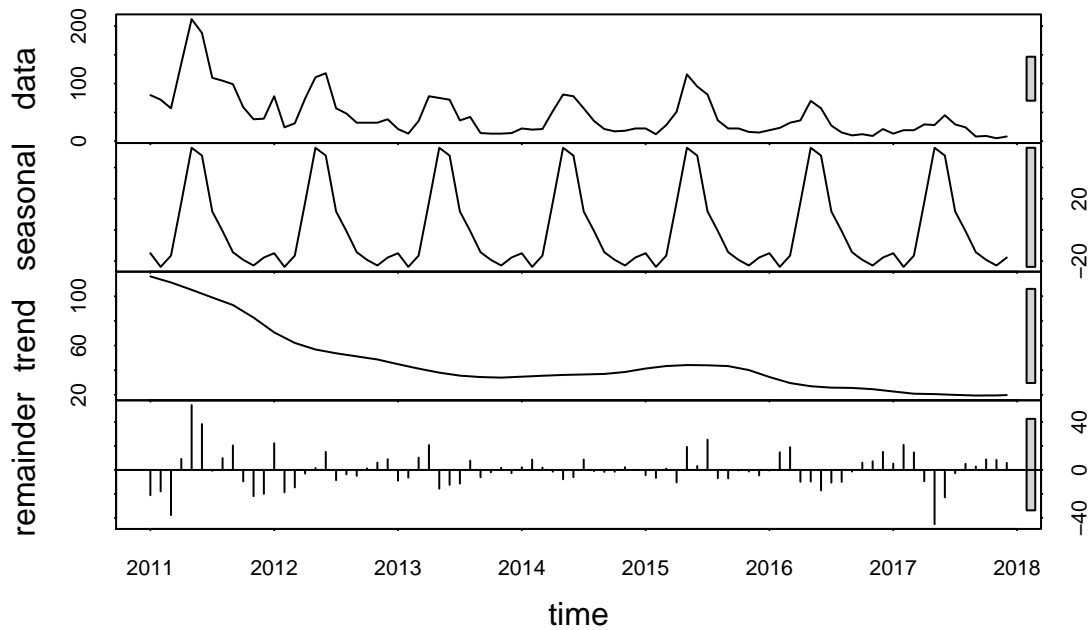

Supplement: S5 Fig — (PDF) [file pone.0226910.s005.pdf]
